# Supplementary figures and images for: Enumerating regulatory T cells in cryopreserved umbilical cord blood samples using FOXP3 methylation specific quantitative PCR
Source: PLoS One. 2020 Oct 23;15(10):e0240190. doi: 10.1371/journal.pone.0240190 (PMC7584164; doi:10.1371/journal.pone.0240190)

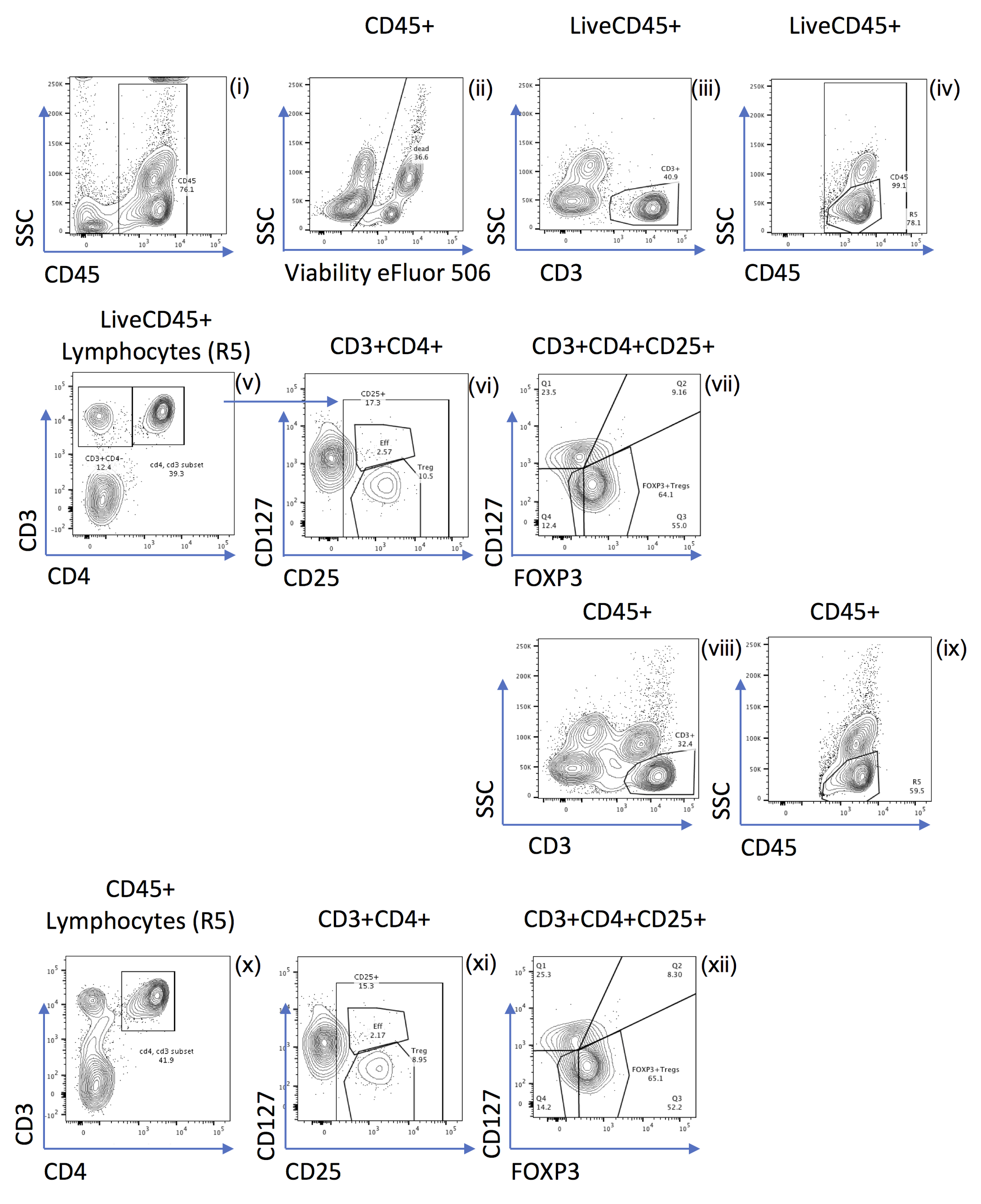

Supplement: S1 Fig — The same gating strategy as applied in Fig 1 but with an example thawed CB segment. Left to right top row; (i) gating CD45+ cells, (ii) excluding dead cells (eFluor506+), (iii) gating SSClow/CD3+ cells and (iv) CD45hiSSClow lymphocytes (R5). Second row, (v) CD3+CD4+ cells are gated from R5 cells and then (vi) effectors (CD127hi) and Tregs (CD127low) CD25+ cells. (vii) gated Tregs from CD127lowFOXP3hi CD25+ cells. (viii–xii); same as (iii–vii) but from total CD45+ cells (i) and without exclusion of dead cells (ii). (TIF) [file pone.0240190.s001.tif]

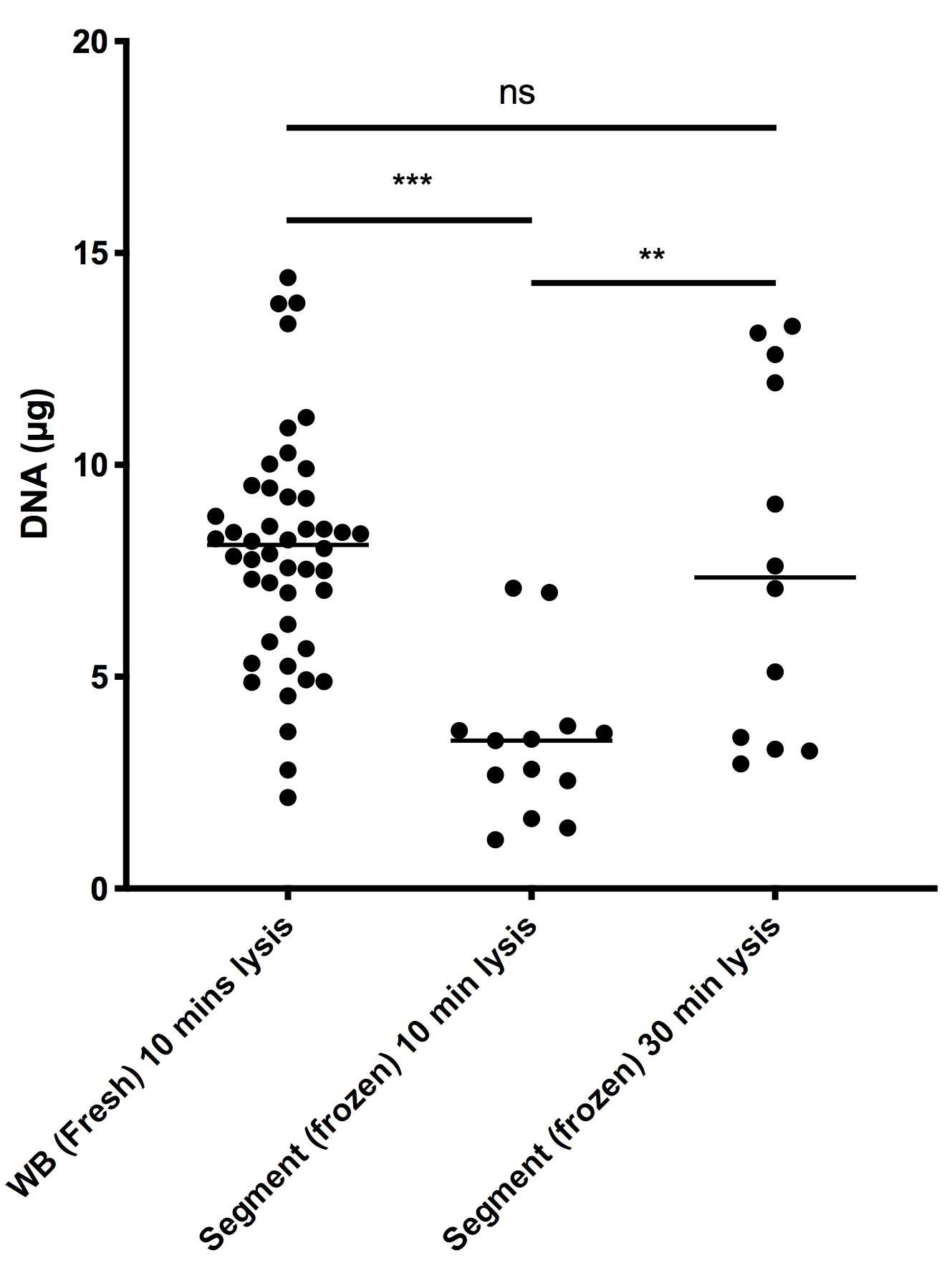

Supplement: S2 Fig — Results of non-parametric unpaired Mann-Whitney U tests are as shown *** = p ≤ 0.001, ** = p ≤ 0.01. (TIF) [file pone.0240190.s002.tif]

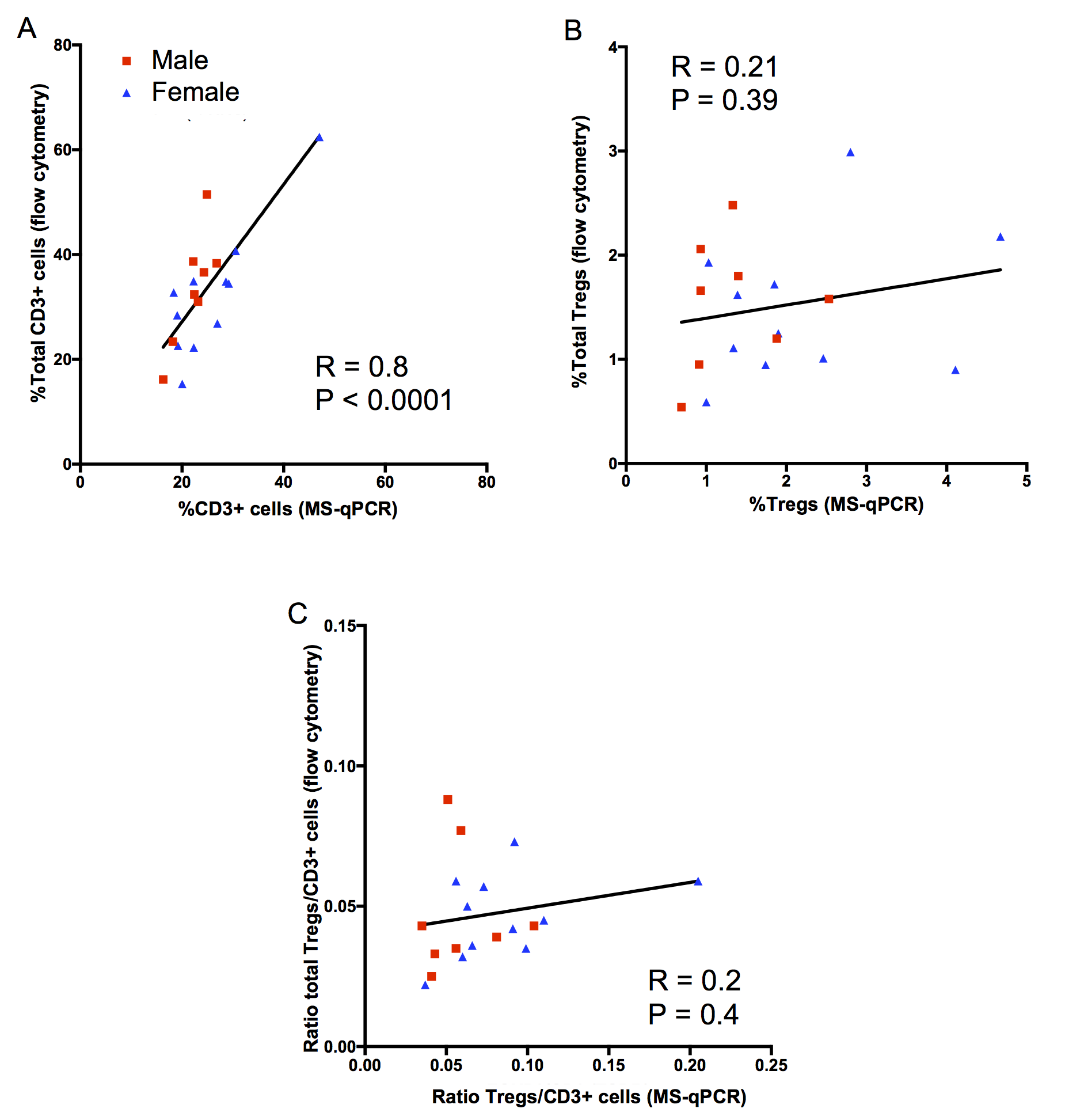

Supplement: S3 Fig — Cellular enumerations, from frozen CB segments, using flow cytometry were compared with epigenetic enumerations performed on the same samples. Male and female derived samples are as indicated. Flow cytometry assessments used total cell gating (gating without the exclusion of dead cells as shown in S1). A; CD3+ cells by flow cytometric or with TcSDR MS-qPCR enumeration. B; Treg by flow or with TSDR MS-qPCR enumeration. C; Ratio of Treg/CD3+ cells using the two methods. (TIF) [file pone.0240190.s003.tif]

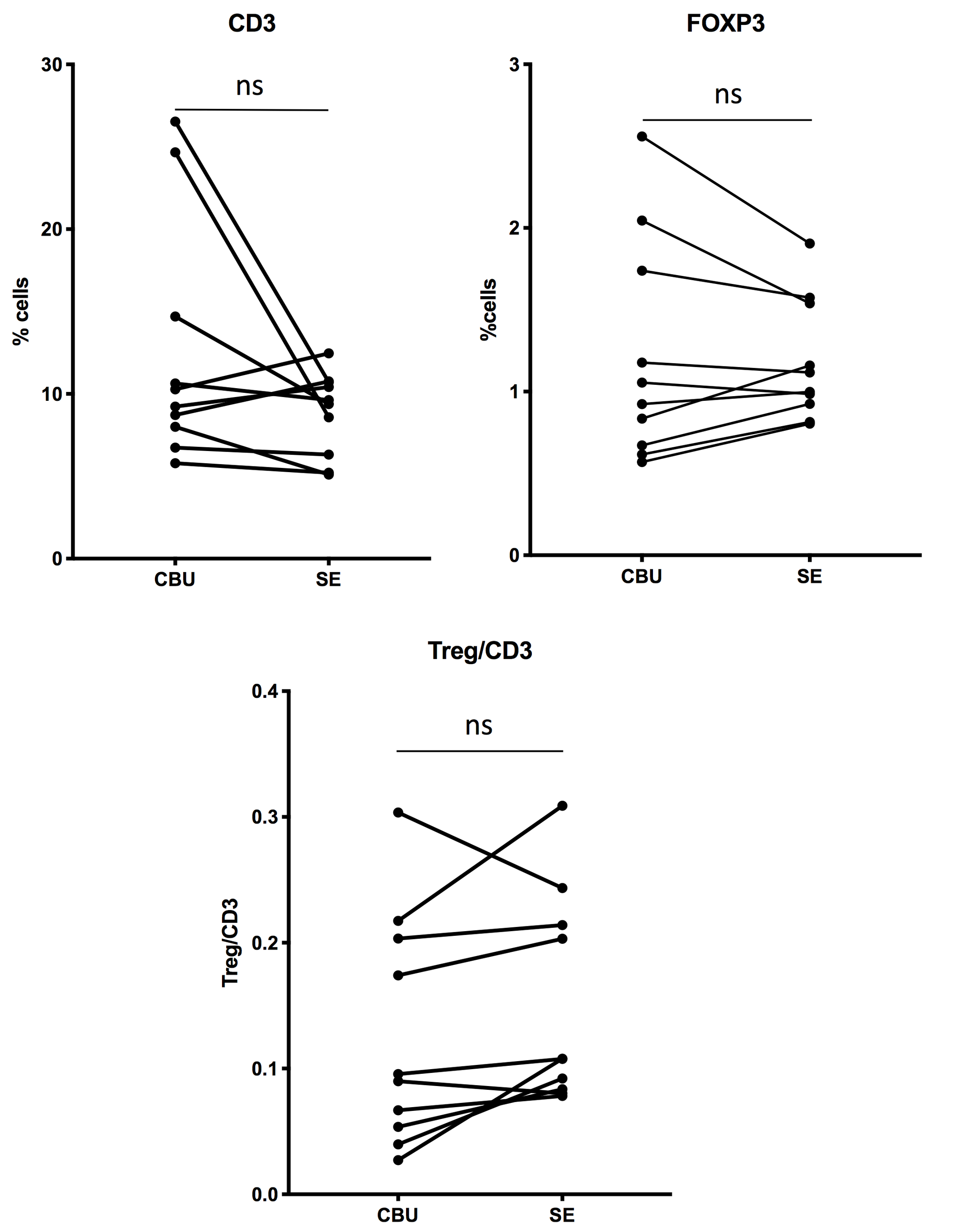

Supplement: S4 Fig — Shown are MS-qPCR based enumerations in fresh samples from whole units (CBU), and paired samples from frozen segments (SE) from the same units. Shown is the results of Wilcoxon tests for paired observations. (TIF) [file pone.0240190.s004.tif]
